# Biofilm Formation Ability of *Arcobacter*-like and *Campylobacter* Strains under Different Conditions and on Food Processing Materials

**DOI:** 10.3390/microorganisms9102017

**Published:** 2021-09-23

**Authors:** David Šilha, Sabina Sirotková, Karolína Švarcová, Leona Hofmeisterová, Květa Koryčanová, Lucie Šilhová

**Affiliations:** Department of Biological and Biochemical Sciences, Faculty of Chemical Technology, University of Pardubice, Studentská 573, 532 10 Pardubice, Czech Republic; toskan@centrum.cz (S.S.); Karolina.Svarcova@student.upce.cz (K.Š.); Leona.Hofmeisterova@student.upce.cz (L.H.); Kveta.Korycanova@upce.cz (K.K.); Lucie.Silhova@atlas.cz (L.Š.)

**Keywords:** *Arcobacter*-like, *Aliarcobacter* spp., *Campylobacter* spp., biofilm formation, abiotic surfaces, temperature condition, food processing materials

## Abstract

*Campylobacter* *jejuni* is the most frequent cause of bacterial gastrointestinal food-borne infection worldwide. The transmission of *Campylobacter* and *Arcobacter*-like species is often made possible by their ability to adhere to various abiotic surfaces. This study is focused on monitoring the biofilm ability of 69 strains of *Campylobacter* spp. and lesser described species of the *Arcobacteraceae* family isolated from food, water, and clinical samples within the Czech Republic. Biofilm formation was monitored and evaluated under an aerobic/microaerophilic atmosphere after cultivation for 24 or 72 h depending on the surface material. An overall higher adhesion ability was observed in arcobacters. A chi-squared test showed no association between the origin of the strains and biofilm activity (*p* > 0.05). *Arcobacter*-like species are able to form biofilms under microaerophilic and aerobic conditions; however, they prefer microaerophilic environments. Biofilm formation has already been demonstrated at refrigerator temperatures (5 °C). Arcobacters also showed higher biofilm formation ability at the temperature of 30 °C. This is in contrast to *Campylobacter* *jejuni* NP 2896, which showed higher biofilm formation ability at temperatures of 5–30 °C. Overall, the results demonstrated the biofilm formation ability of many strains, which poses a considerable risk to the food industry, medical practice, and human health.

## 1. Introduction

Biofilms are microbial communities growing on the surface or interface of materials. Cells are “interconnected” in a biofilm, usually via a matrix formed of extracellular polymeric substances (EPS) that they produce themselves [1]. The biofilm structure provides certain benefits to the microorganisms [2]. Bacteria living in biofilms exhibit increased resistance to host defense mechanisms and up to 1000 times higher natural resistance, e.g., to antibiotics [3,4]. The current trend is also to monitor the influence of many natural substances on the formation of microbial biofilms [5,6]. In nature, we often observe multi-species biofilms created on abiotic or biotic surfaces [7]. Biofilm formation requires a special type of signaling, known as quorum sensing (QS; also called density sensing) among the microorganism cells [8]. QS controls a variety of physiological behaviors of Gram-positive and Gram-negative bacteria and there are a variety of mechanisms and signal molecules used in QS to transmit information [9]. Biofilm formation comprises several stages, namely, the initial attachment to the surface, micro-colony formation, maturation, and detachment (dispersion) of biofilm [8]. The biofilm formation itself can be reduced, for example, by reducing the adhesion of microorganisms, or we must subsequently address the eradication of the biofilm formed [2]. However, biofilm formation is also largely dependent on various environmental influences and mechanisms affecting microbial cell gene expression [10]. Adhesion to the surfaces is a fundamental step in biofilm formation and leads to significant changes in cell metabolism, mainly to the expression of EPS and various microbial surface components, which recognize adhesive matrix molecules [11].

Biofilms formed on food processing surfaces are of great concern. The hygiene of these surfaces affects the quality of food products [12]. Biofilms constituted by pathogenic bacteria can result in product contamination that may lead to health problems. In fact, biofilms are known to be frequent sources of infections and many foodborne outbreaks have been associated with them [13,14]. A number of methods are used to demonstrate biofilm formation. The gold standard is the widely used Christensen phenotypic colorimetric method that uses crystal violet to color the biofilm layer. Genotypic methods that demonstrate the presence of genes responsible for biofilm formation are also useful [15,16,17].

It is estimated that over 80 % of the bacterial population on Earth is capable of biofilm formation under certain conditions [18]. Biofilm-forming bacteria also include *Arcobacter*-like and *Campylobacter* strains [17,19,20]. These are Gram-negative bacteria that grow at 30–37 °C, or even up to 42 °C in the case of campylobacters. The basic difference between arcobacters and the *Campylobacter* spp. is the ability of arcobacters to grow at lower temperatures and in both aerobic and microaerophilic environments. In many studies, the occurrence of these bacteria has been reported mainly in food and water, but also in the environment [21,22]. Recently, based on a wide comparative genomic analysis, reclassification and a new *Arcobacteraceae* family was proposed [23]. Further, the taxonomy of the genus *Arcobacter* has been revisited using the core genome of 57 strains, a multilocus sequence analysis with 13 housekeeping genes, and genomic indexes like average nucleotide identity (ANI), in silico DNA–DNA hybridization (*is*DDH), average amino acid identity (AAI), percentage of conserved proteins, etc. According to results, genomic and phylogenetic groups were delimited and six different genera, including *Aliarcobacter* gen. nov., were proposed. This genus comprises seven species, including *Aliarcobacter butzleri* comb. nov., *Aliarcobacter cryaerophilus* comb. nov., *Aliarcobacter skirrowii* comb. nov., etc. [24]. These microorganisms can cause gastroenteritis and other human and animal diseases. The association of arcobacters with human disease has been demonstrated for *A. butzleri*, *A. cryaerophilus*, *A. skirrowii* and *A. thereius* [25,26]. For these bacteria, survival in the form of a biofilm is essential, as they can thus better colonize hosts or contaminate food factory environments [22,27].

The aim of this study was to monitor the biofilm formation of many *Arcobacter*-like and *Campylobacter* strains depending on the conditions of the cultivation atmosphere, the cultivation time, and also on the type of abiotic surface in terms of bacterial cell adherence. For selected strains, biofilm formation was monitored depending on the temperature and type of material surface used in the industry. Collection strains were included in the study, some of which do not have sufficient evidence in the literature about their ability to form biofilms. However, many *Arcobacter*-like strains isolated within the Czech Republic from food and water samples were also included. Moreover, as far as we know, this study is one of the few that also includes clinical isolates of both *Campylobacter* and *Arcobacter*-like species for biofilm formation testing.

## 2. Materials and Methods

### 2.1. Bacterial Strains and Identification by m-PCR and 16S RNA-RFLP

A total of 69 isolates and collection strains of the *Arcobacter*-like bacteria and *Campylobacter* spp. were used to determine their biofilm formation on different surfaces and under different culture conditions. Bacterial strains were used from the Czech Collection of Microorganisms (CCM), Culture Collection University of Göteborg (CCUG), Belgian Co-ordinated Collections of Microorganisms (LMG), or strains isolated at the University of Pardubice (UPa) according to a previously described protocol [21]. Furthermore, some clinical strains were obtained from Pardubice Region Hospital, a.s. (Litomyšl Hospital, Pardubice Hospital) and Náchod Regional Hospital, a.s. (Náchod Hospital). Before testing, cultures were grown on tryptone soya agar (TSA; HiMedia, Mumbai, India) either for 48 h at 30 °C under aerobic conditions (*Arcobacter*-like strains) or for 48 h at 42 °C under microaerophilic conditions (*Campylobacter* spp.). Cells were harvested and suspended in physiological saline to a 0.5 McFarland scale (ca 1.5 × 10^8^ CFU/mL). The suspension of cells was then diluted to an appropriate density before testing. For the verification of the exact number of cells in the prepared cell suspension, a presumptive density of 10^3^ CFU/mL was counted on TSA.

All strains were identified using the m-PCR method [28]. Briefly, PCR reactions were performed in a reaction mixture (50 µL final volume) composed of PCR water, 5 µL of 10× PCR buffer, 1.5U *Taq* polymerase and a deoxynucleotide triphosphate mixture at a final concentration of 0.2 mM each. The mixture also contained 1.5 mmol of MgCl_2_ and 50 pmol of each primer (ButR, SkiR, TherR, CibR, ArcoF, GyrasF, and GyrasR). Prior to cycling, samples were heated to 94 °C for 3 min. The PCR assay involved 30 cycles of denaturation (94 °C, 45 s), primer annealing (58 °C, 45 s), and chain extension (72 °C, 2 min) with a final elongation step at 72 °C for 5 min. PCR products were separated in 2% agarose gel in 0.5× TBE buffer at 120 V for 2 h. Gels were stained with ethidium bromide (1 µg/mL).

Due to the possibility of distinguishing *A. cryaerophilus* strains into 1A and 1B and also to increase the reliability of identification based on previous knowledge, all strains were subjected to identification according to the 16S rRNA-RFLP protocol [29]. Genomic DNA was used as a template for the PCR amplification of a 1026 bp region of the 16S rRNA gene, as previously described [30]. Amplicons were digested with *Mse*I or *Mn*II/*Bfa*I (BioLabs New England, Ipswich, MA, USA) in a 25 µL final volume consisting of 0.5 µL of the PCR amplicons, 5 U of the enzyme (BioLabs New England, Ipswich, MA, USA), 2.5 µL of 10× CutSmart buffer (BioLabs New England, Ipswich, MA, USA), and PCR water. The reaction mixture was digested at 37 °C for 15 min (*Mse*I or *Mn*II) or 1 h (*Bfa*I). Enzyme activity was inhibited at 65 °C for 20 min (*Mse*I, *Mn*II) or at 80 °C for 20 min (*Bfa*I) in accordance with the manufacturer’s instructions. The digested products were separated on 3% agarose gels in 1× TBE buffer at 100 V for 120 min. A 50 bp DNA size marker (BiotechRabbit, Berlin, Germany) was used to confirm the expected amplicon size of each target gene. Gels were stained with ethidium bromide (1 µg/mL) and later photographed on a UV transilluminator.

### 2.2. Determination of Biofilm Formation in Microtiter Plate at Various Conditions

Biofilm formation was monitored in flat-bottomed microtiter plates (SPL Life Sciences, Pocheon-si, South Korea) as previously described [31,32] with some modifications. Briefly, 100 µL of cell suspension (10^7^ CFU/mL) in brain heart infusion broth (BHI; Himedia, Mumbai, India) was inoculated into 96-well flat-bottomed microtiter polystyrene plates (SPL Live Sciences Co., Ltd., Korea). After incubation under specific conditions, the wells were rinsed thoroughly five times with sterile distilled water and dried. Biofilm fixation was performed with 2% sodium acetate (15 min) and biofilm-forming cells were stained with 100 µL of 1% crystal violet (Sigma-Aldrich, St. Louis, MO, USA). After 15 min of staining, the plate was repeatedly washed and dried. Thereafter, the biofilm-associated violet was solubilized with 96% ethanol and the absorbance (OD) of the solution was measured in a new plate at 595 nm (Infinite M200, Tecan, Männedorf, Switzerland). There were 24 wells in each experiment, and the experiments were independently repeated three times.

The biofilm formation level of the *Arcobacter*-like strains was categorized according to the classification system previously described [32] as non-adherent (N; OD ≤ OD_C_) or weakly (W; OD_C_ < OD ≤ 2 × OD_C_), moderately (M; 2 × OD_C_ < OD ≤ 4 × OD_C_), or strongly adherent (S; 4 × OD_C_ < OD), where OD_C_ (cut-off OD) is defined as three standard deviations above the mean OD of the negative control (blank value). The measured and calculated OD/OD_C_ (0.111/0.120) values were the same for all measurements.

### 2.3. Determination of Biofilm Formation in Glass Tube at Various Conditions

Biofilm formation was monitored in glass tubes as previously described by [15] with some modifications. Briefly, 2 mL of cell suspension (10^7^ CFU/mL) in brain heart infusion broth (BHI; Himedia, Mumbai, India) was inoculated into sterile borosilicate glass tubes. After incubation under specific conditions, the tubes were rinsed thoroughly five times with sterile distilled water and dried. The tubes were stained with 1% crystal violet and then incubated at room temperature for 15 min. The tubes were washed three times with distilled water and dried completely at 37 °C.

Evaluation of biofilm formation was performed visually by comparing with a weakly adherent *E. coli* CCM 3954 and a strongly adherent *S. aureus* CCM 4224. All experiments were repeated at least in triplicate and independently repeated three times. The results are recorded as non-adherent (N), weakly (W), moderately (M), or strongly (S) adherent.

### 2.4. Biofilm Formation of Selected Adherent Strains on Food Processing Materials

Biofilm formation on food processing materials was monitored as previously described with some modifications [33]. Biofilm formation ability of selected strains (*A. defluvii* LMG 25694, *A. butzleri* UPa 2013/3, *A. cryaerophilus* 1B UPa 2012/1, *C. jejuni* NP 2896) was evaluated after incubation in different liquid environments at 5, 10, 25, 30, and 42 °C for 24 h under aerobic atmosphere. Materials intended for food the industry or kitchen food preparation were chosen for further testing—stainless steel (type 304 finish 2b; Terapol, Prague, Czech Republic), plastic (PE-LD, plastic cutting board; IKEA, Sweden), and glass (microscope slides; Corning Glass Works, Corning, NY, USA). Material coupons (4 cm × 1 cm) were first cleaned using an ultra-fine brush and liquid detergent. Coupons were rinsed in distilled water before being soaked in 70% ethanol. The coupons were allowed to air dry and then autoclaved before use. The biofilm sample was prepared by culturing 1 mL of bacterial suspension at a cell density of 10^8^ CFU/mL in 9 mL of tryptone soya broth (TSB; Himedie, Mumbai, India), BHI broth, or peptone water with 0.9% NaCl. After incubation, the coupon was removed from the culture medium and rinsed gently but repeatedly with sterile distilled water to remove planktonic cells. Biofilm cells were scraped off with a cotton swab, which was then transferred to a tube with 10 mL PBS. After intensive shaking of the swab (vortex, 2 min) and sonication (48 kHz, 3 min), the amount of biofilm-forming cells was monitored by culture. All experiments were repeated in at least triplicate.

### 2.5. Statistical Analysis

Statistical analysis was performed with Excel 2010 (Microsoft, Redmond, WA, USA) and Statistica 12 (StatSoft, Tulsa, OK, USA). Extreme values were tested with the Dean-Dixon test, and all remoteness values were excluded with 95% probability. Median and standard deviation were determined from the remaining values. A possible source of error resulting from insufficient dye washing resulting in an increase in absorbance was also considered, and absorbance values that were too high compared to other measured values were excluded.

Statistical significance was determined by chi-squared test. A *p* value of <0.05 was considered to be statistically significant. The calculated standard deviations of the measurements are not shown in Table 1, Table 2, Table 3 and Table 4, but these values did not exceed 5% in any experiment.

## 3. Results

### 3.1. 16S rRNA-RFLP and m-PCR Identification of Strains

A total of 51 *Arcobacter*-like isolates were recovered from food, water samples, or clinical strains. All isolates were successfully identified as *A. butzleri* or *A. cryaerophilus* using both mPCR and 16S rRNA-RFLP methods and used for further testing. Two subgroups of *A. cryaerophilus*, 1A and 1B, were further distinguished based on the restriction profiles attained with 16S rRNA-RFLP. Other strains were obtained from collections of microorganisms as properly identified strains.

### 3.2. Biofilm Formation of Arcobacter-like Bacteria under Different Conditions

#### 3.2.1. Biofilm Formation on Plastic (Polystyrene) Material

The biofilm activity of 59 *Arcobacter*-like strains was studied under various culture conditions on a polystyrene surface (Table 1, Table 2 and Table 3). The obtained data reveal that the strains tested differ significantly in the intensity of their biofilm formation. However, an association between strain origin and biofilm activity was not observed at a statistically significant level (*p* > 0.05). The categorization of biofilm activity was influenced by the culture atmosphere in a total of 31 strains (52.5%) of arcobacters. Of this number, 27 strains (87.1%) had significantly increased biofilm activity under microaerophilic culture conditions. This can be explained by the fact that arcobacters usually grow under aerobic conditions and microaerophilic conditions subject cells to an increase in stress, to which they may respond by increasing biofilm formation. However, in five strains (8.5%), increased biofilm formation was observed under aerobic culture conditions. For example, in *A. butzleri* UPa 39-3, increased biofilm production under aerobic conditions was observed after both 24 and 72 h of cultivation.

The other monitored factor influencing biofilm formation was exposure time. According to results in Table 1, Table 2 and Table 3, 18.6/11.9% of isolates were categorized as non-adherent, 78.0/79.7% as weakly adherent, and 3.4/8.5% were included in the category of moderately adherent under aerobic/microaerophilic conditions after 24 h of exposure. After 72 h of exposure, 25.4/5.1% of isolates were categorized as non-adherent, 71.2/88.1% as weakly adherent, and 3.4/5.1% were included in the category of moderately adherent under aerobic/microaerophilic conditions. According to the measured absorbances, the biofilm-forming ability of weakly adherent isolates ranged from 0.121 to 0.240, moderately adherent isolates exhibited a range of biofilm ability varying from 0.241 to 0.418. Only one strain (1.7%) was rated as strongly adherent under microaerophilic conditions and after 72 h of exposure.

As was mentioned above, an increased amount of biofilm was observed in some strains after prolonged cultivation. This was most evident in *A. defluvii* LMG 25694. In this strain, the amount of biofilm detected increased by more than 80.0% after 72 h of cultivation under aerobic conditions (see Table 3). The same was observed for the clinical isolate of *A. butzleri* UPa 39-3 (A_595_A24h_ = 0.143 ± 0.006 and A_595_M72h_ = 0.242 ± 0.017). However, for example in the *A. butzleri* UPa 2015/7 strain, a reduced amount of detected biofilm was recorded after 72 h of cultivation (A_595_A24h_ = 0.241 ± 0.011; and A_595_M72h_ = 0.120 ± 0.003). However, in most strains approximately the same biofilm production was observed after exposure for 24 and 72 h under otherwise identical conditions.

#### 3.2.2. Biofilm Formation on Glass (Borosilicate) Material

The biofilm activity of 59 *Arcobacter*-like strains was studied under various culture conditions on borosilicate surfaces (Table 1, Table 2 and Table 3). The obtained results show significantly different intensities of biofilm produced. However, an association between strain origin and biofilm activity was not observed at a statistically significant level (*p* > 0.05). The vast majority of strains (58; 98.3%) were confirmed to be capable of adhesion to the borosilicate surface under at least some experimental conditions. The categorization of biofilm activity was influenced by the cultivation atmosphere in a total of 43 strains (72.9%) of arcobacters. Of these, 38 strains (88.4%) had increased biofilm activity under aerobic cultivation conditions.

According to the results presented in Table 1, Table 2 and Table 3, 27.1/32.2% of isolates were categorized as non-adherent, 30.5/45.8% as weakly adherent, 40.7/20.3% as moderately adherent and 1.7/1.7% were included in the category of strongly adherent under aerobic/microaerophilic conditions after 24 h of exposure. After 72 h of exposure, 8.5/23.7% of isolates were categorized as non-adherent, 20.3/49.2% as weakly adherent, 67.8/25.4% as moderately adherent, and 3.4/1.7% were included in the category of moderately adherent under aerobic/microaerophilic conditions. Higher biofilm production on glass under an aerobic atmosphere was observed in many strains. This applies to strains across the species of interest (e.g., *A. cryaerophilus* 1B UPa 2012/1, *A. butzleri* UPa 2012/3, *A. lanthieri* LMG 28517, etc.).

Further, greater production of arcobacter biofilms on the borosilicate surface was confirmed after a longer cultivation period (Table 1, Table 2 and Table 3). The strains of *A. skirrowii* LMG 6621 and *A. thereius* LMG 24488 did not form a biofilm after 24 h of cultivation on glass, but after prolonged cultivation the biofilm formation was relatively high. A similar phenomenon was observed with *A. butzleri* UPa 2015/13, *A. butzleri* UPa 2015/15, *A. butzleri* UPa 2015/20, *A. cryaerophilus* 1B UPa 2014/58A, and *A. cryaerophilus* 1B UPa 130 (after a longer exposure period, higher biofilm production was observed under both aerobic and microaerophilic conditions).

Our results showed that incubation under an aerobic or microaerophilic atmosphere may influence biofilm formation ability on abiotic surfaces. Atmospheric conditions could therefore be relevant to the food-based transmission of *Arcobacter*-like bacteria.

### 3.3. Biofilm Formation of Campylobacter spp. under Different Conditions

#### 3.3.1. Biofilm Formation on Plastic (Polystyrene) Material

According to Table 4, 0.0/40.0% of isolates were categorized as non-adherent, and 100.0/60.0% were included in the category of weakly adherent under aerobic/microaerophilic conditions after 24 h of exposure. After 72 h of exposure, 100.0/70.0% of isolates were categorized as non-adherent, and 0.0/30.0% were included in the category of weakly adherent under aerobic/microaerophilic conditions. The measured absorbance—biofilm formation ability of weakly adherent isolates ranged from 0.121 to 0.132.

In 10 strains (100.0%) of campylobacter, the ability to adhere to the polystyrene surface was confirmed under at least some experimental conditions. The categorization of biofilm activity was influenced by the culture atmosphere in 7 strains (70.0%). Of these, 4 strains (57.1%) exhibited increased biofilm activity under aerobic culture conditions.

All campylobacters exhibited adherence under aerobic conditions after 24 h of exposure. The results obtained probably also indicate in this case that the campylobacters, which usually grow microaerophilically, only exhibit higher biofilm production under unfavorable aerobic cultivation conditions. However, an increase in the amount of biofilm after microaerophilic cultivation was observed in some strains, e.g., in the *C. coli* NP 2395 strain (A_595_M72h_ = 0.125 ± 0.007; and A_595_A72h_ = 0.118 ± 0.003).

According to our results, no biofilm formation was observed in most strains (70.0%) after 72 h of exposure. The biofilm was only detected after 72 h of exposure in the *C. jejuni* NS 3800, *C. jejuni* NS 4088, and *C. coli* NP 2395 strains. The cultivation time is probably so long that the biofilm structure is already dying and gradually peeling off the surface of the material.

#### 3.3.2. Biofilm Formation on Glass (Borosilicate) Material

Most *Campylobacter* strains (90.0%) were confirmed to be able to adhere to the borosilicate surface at least under some experimental conditions. The categorization of biofilm activity was influenced by the cultivation atmosphere in seven strains (70.0%) of campylobacters. Of these, four strains (57.1%) exhibited increased adherence under microaerophilic conditions of cultivation. According to Table 4, 30.0/70.0% of isolates were categorized as non-adherent, and 60.0/20.0% were included in the category of weakly adherent under aerobic/microaerophilic conditions after 24 h of exposure. After 72 h of exposure, 80.0/50.0% of isolates were categorized as non-adherent, and 10.0/40.0% were included in the category of weakly adherent under aerobic/microaerophilic conditions. Overall, the highest biofilm production on the borosilicate surface was observed in the *C. jejuni* CCM 6214 strain under all experimental conditions.

After 24 h of aerobic cultivation, only weak biofilm formation was observed in campylobacters; in *C. jejuni* NS3668, *C. jejuni* NS3800 and *C. jejuni* NS4088, no biofilm formation was detected at all under the given conditions. After prolonged cultivation (72 h) in an aerobic environment, 80.0% of strains no longer exhibited biofilm formation (with the exception of *C. jejuni* CCM 6214 and *C. jejuni* NS 3668).

### 3.4. Biofilm Formation on Food Processing Materials under Various Conditions

**Figure 1:**
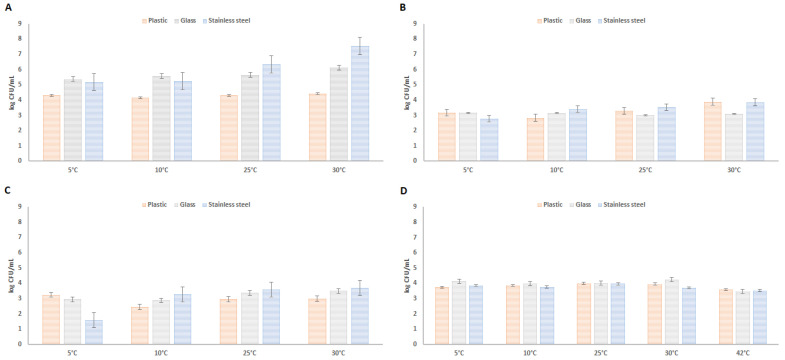
Biofilm formation of *Arcobacter*-like ((**A**)—*A. defluvii* LMG 25694; (**B**)—*A. cryaerophilus* 1B UPa 2012/1; (**C**)—*A. butzleri* UPa 2013/3) and *Campylobacter* ((**D**)—*C. jejuni* NP 2896) strains on food processing materials at various temperatures and aerobic conditions.

Based on previous testing, *Arcobacter*-like and *Campylobacter* strains that showed moderate to strong biofilm formation activity were selected for further monitoring of biofilm formation on materials used in the food industry or in food processing (glass, plastic, stainless steel). The results of this testing are presented on Figure 1. The results show that the highest biofilm activity was observed in the case of *A. defluvii* LMG 25694. Increasing biofilm activity was also observed in this strain with increasing culture temperature. The highest biofilm formation in the case of this strain was observed after cultivation at 30 °C on stainless steel. The lowest amount of biofilm was observed in the strain *A. butzleri* UPa 2013/3 after cultivation at 5 °C on stainless steel, but at higher cultivation temperatures the detected amount of biofilm in this strain was highest on stainless steel. Relatively balanced biofilm formation was observed in *Campylobacter jejuni* NP 2896 strain. There were no significant differences in the formation of the amount of biofilm produced on individual materials in this strain. Nevertheless, it can be stated that lower biofilm formation was observed at the optimal culture temperature of *Campylobacter* (42 °C), compared to lower temperatures.

## 4. Discussion

The correct identification of microbial isolates is the basis for further experimental work. Several molecular methods have been designed for *Arcobacter* identification at the species level. The most globally used method is mPCR developed by Houf et al., although this method produces misidentification of some recently described species. However, the PCR protocol described by Douidah et al. is able to distinguish more species and provide more reliable results [34] and this protocol is often used to identify arcobacters isolated from many samples, including clinical isolates [25]. According to our results, this mPCR method is suitable for the identification of arcobacters, especially in view of the lower possibility of confusion of identified species. However, 16S rRNA-RFLP is able to help with identification of arcobacters, including recently described ones. This method is reproducible, reliable, simple, fast, and reasonably inexpensive. The technique is highly applicable for investigations of the prevalence of *Arcobacter* in a variety of food products, water, or other samples [29]. Based on the results of identification using 16S rRNA-RFLP, it was also possible to distinguish the *A. cryaerophilus* isolates included in this study into subgroups 1A and 1B.

The genera *Campylobacter* and *Arcobacter* have become increasingly important in recent years because their members have been considered emergent enteropathogens and potential zoonotic agents [34]. *Arcobacter*-like bacteria, mostly *A. butzleri*, are frequently isolated from products of animal origin with the highest prevalence occurring in poultry products [19]. However, the occurrence of these bacteria might be underestimated due to no strict methodology for the isolation and identification of these bacteria. Biofilm formation ability is among the factors influencing bacterial virulence, antibiotic resistance, etc. The biofilm formation of arcobacters and campylobacters has been previously described, for example on stainless steel, copper, glass, and plastic surfaces [17,19,35,36,37,38,39,40]. However, arcobacters are generally described in the literature as weak biofilm producers [38]. Similarly, many studies indicate that campylobacters are not strong biofilm producers [17,39,40,41], which is consistent with the results of our study. According to the obtained results, it can be stated that the *Campylobacter* species exhibited slightly higher biofilm production in their natural microaerophilic environment, which has also been reported elsewhere [17].

In some studies, higher biofilm activity has been reported in strains isolated from meat [38], which was not shown to be statistically significant (*p* > 0.05) in our results. To our knowledge, relatively recently taxonomically incorporated, and hence substantially less described, *Arcobacter*-like species have not been included in any similar studies yet. In this respect, positive biofilm formation on polystyrene surfaces has been found, for example, in *A. skirrowii* LMG 6621, *A. thereius* LMG 24488, *A. lanthieri* LMG 28517 and in particular *A. defluvii* LMG 25694, in which the highest biofilm formation was found under all experimental conditions. However, it is apparent that biofilm formation is a variable capability and, in some circumstances, a strain may not produce a biofilm at all, even though it has been previously described as biofilm-positive strain. Our results show that the biofilm formation of strains isolated from a real environment does not differ significantly (*p* > 0.05) from the collection type strains, as evidenced also in a previous study [42]. However, another previous study reported that *Arcobacter* strains isolated from the environment exhibited a greater ability to form biofilms compared to collection strains [19].

Significant external factors such as environmental conditions or surface properties also influence biofilm formation. As has been previously mentioned, it is not generally possible to define which culture conditions are or are not suitable for biofilm formation [37,38]. The availability of nutrients or the composition of the culture medium used also has a significant influence on biofilm formation. For example, TSB medium [43] or BHI broth [44] are often used for testing. In contrast, Mueller-Hinton broth is not nutritionally well suited for these purposes [38].

The ability to adhere to the polystyrene surface of the microtiter plates was confirmed in at least some experimental conditions in all 59 (100.0%) of the studied *Arcobacter*-like strains. Some studies report the biofilm formation ability of a high number of *Arcobacter* strains [19,35]; in other studies, this was confirmed in only 21.4% of the studied strains [38]. Obviously, the type of material significantly affects the adherence and biofilm formation of arcobacters [37,38]. According to our results, *A. butzleri* UPa 2015/12 strain was rated as non-adherent on all materials and under all conditions. However, adherence-capable strains were generally rated as stronger biofilm producers than the strains that adhered to plastic surfaces only (see Table 1, Table 2 and Table 3).

It has been confirmed in the past that biofilm activity is influenced by the culture atmosphere [19,38]. An aerobic environment may promote the adhesion properties of some *Arcobacter*-like strains, while other strains are stimulated by microaerophilic conditions [19]. Earlier studies also reached similar conclusions in the case of *Campylobacter*, suggesting that the unnatural aerobic atmosphere promotes the formation of a biofilm structure [17,27]. Based on our results, higher biofilm production under an aerobic atmosphere was recorded; however, some strains shown increase in biofilm formation in microaerophilic atmospheres.

Exposure time has a significant effect on biofilm formation. However, it cannot be stated that a longer cultivation time always means a higher amount of detected biofilm on a given material. This is probably related to different cell tolerance to environmental influences, the susceptibility of the biofilm layer to peeling, etc. It has been reported in the past that *A. butzleri* and *A. cryaerophilus* produced the most biofilm after 24 h of cultivation and then there was no significant change in the amount of biofilm detected [35] that is consistent with our results. However, some authors also mention that the amount of produced biofilm increases with increasing cultivation time [35]. In an earlier study, it was confirmed that campylobacters are still able to form a biofilm after 48 h of cultivation [37]. For many strains included in our study, no biofilm formation was observed after 72 h of exposure, which may be related to the peeling of the formed “integral” layer of biofilm on the given material.

When monitoring the biofilm formation on coupons made of various materials, it is a simulation of more realistic conditions that we encounter in food processing. Stainless steel or plastic material is widely used in the food industry because it exhibits some of the most suitable characteristics of the construction materials used for food equipment [38]. The ability of *Arcobacter* and *Campylobacter* spp. to adhere to these inert surfaces in the form of biofilms has previously been described [35,41]. The first study describing the adhesion of *A. butzleri* to stainless steel was published in 2002 [45]. Subsequently, another study confirmed the formation of a biofilm on stainless steel in just 14.3% of studied strains of *A. butzleri* [35]. Therefore, the findings of the current studies provide confirmation of the ability of *A. butzleri* to adhere to extensively used materials in the food industry [37,38]. However, most studies present the results of arcobacters biofilm formation at 30 °C. The results of our study show that arcobacters are capable of relatively large biofilm production, even at lower temperatures such as refrigeration temperatures. Most studies on *C. jejuni* biofilms have thus far been carried out at 37 °C [46]. However, Dykes et al. found that *C. jejuni* grown as planktonic cells and as biofilm cells survived longer at lower temperatures (4 °C and 10 °C) in comparison with higher temperatures (25 °C and 37 °C) under stress conditions [47]. While most studies on biofilm formation by *C. jejuni* have been carried out under microaerobic conditions, some of them have been undertaken to examine biofilm formation by *C. jejuni* under aerobic conditions. The biofilm formation of *C. jejuni* is often evaluated to be more intense under aerobic conditions [27]. This can be explained by higher biofilm production in response to oxidative stress. Significant biofilm formation of *Campylobacter* spp. may be of concern, namely even at low temperatures and for a relatively short time.

Even if consumers expect to acquire healthy and safe food products, pathogenic bacteria can be present in a variety of food products. The persistence of pathogenic bacteria in food processing environments leads to food-associated infections. Foodborne illnesses are a growing public health problem worldwide, and their prevention is the main objective of food safety. In this respect, the prevention of cross-contamination is a key factor. However, further investigations are needed to verify the effect of biofilm formation in the spread of pathogens in the food industry.

## 5. Conclusions

This study provides information on the biofilm activity of many *Arcobacter*-like strains and *Campylobacter* spp. isolated from the environment, food or clinical samples collected within the Czech Republic. The results of the study confirm the ability to form a biofilm in almost all the strains involved in this study. However, the resulting biofilm formation is variable and dependent on many environmental factors, especially the atmosphere and the length of the cultivation. Higher adhesion was observed in conditions less favorable for the survival of planktonic forms of these bacteria—under microaerophilic or aerobic conditions in the case of *Arcobacter* spp. or *Campylobacter* spp., respectively.

As far as we know, this is the first study describing biofilm formation in such a large set of strains, including representatives of relatively recently described *Arcobacter*-like species. The study also provides an important insight into the ability of many clinical isolates of *Arcobacter* and *Campylobacter* to adhere to surfaces and form a biofilm. According to our results, the selected strains were able to form a biofilm on food processing materials, even more so at lower temperatures for *C. jejuni* NP 2896.

These data contribute to the understanding of the survival and persistence of these bacteria in their environment, their potential virulence, and relevance as potential pathogens in the food chain or healthcare. It is important to monitor the biofilm formation capability of these bacteria, as they are responsible for a significant percentage of human alimentary infections. Ideally, conditions should be set to prevent the formation of biofilm structures. Subsequent studies of the options for influencing the formation of a biofilm would have great benefits in both the food industry and healthcare.

## Figures and Tables

**Table 1:** Biofilm formation of *A. butzleri* strains on plastic/glass material at 30 °C.

Strains	Exposure for 24 h		Exposure for 72 h	
	Aerobic	Microaerophilic	Aerobic	Microaerophilic
*A. butzleri* UPa 2012/3 *^A^*	W (0.124)/M	W (0.123)/W	W (0.156)/M	W (0.125)/W
*A. butzleri* UPa 2013/3 *^B^*	W (0.173)/M	M (0.262)/M	W (0.129)/M	W (0.136)/N
*A. butzleri* UPa 2013/10 *^A^*	N (0.112)/W	N (0.116)/W	N (0.113)/M	W (0.125)/M
*A. butzleri* UPa 2013/15 *^A^*	N (0.116)/M	W (0.121)/N	W (0.140)/M	W (0.142)/W
*A. butzleri* UPa 2013/30 *^B^*	W (0.149)/M	W (0.178)/M	W (0.124)/W	W (0.137)/M
*A. butzleri* UPa 2013/31 *^B^*	W (0.121)/M	W (0.126)/M	W (0.126)/W	W (0.132)/W
*A. butzleri* UPa 2013/32 *^B^*	W (0.141)/W	W (0.139)/N	W (0.123)/W	W (0.123)/W
*A. butzleri* UPa 2013/33 *^B^*	N (0.112)/W	N (0.117)/W	W (0.122)/N	W (0.127)/N
*A. butzleri* UPa 2013/36 *^A^*	W (0.121)/W	W (0.130)/W	N (0.120)/M	W (0.129)/W
*A. butzleri* UPa 2013/37 *^B^*	W (0.181)/M	W (0.207)/M	W (0.124)/M	W (0.135)/M
*A. butzleri* UPa 2014/51 *^B^*	W (0.123)/W	W (0.122)/N	W (0.125)/M	W (0.122)/W
*A. butzleri* UPa 2014/54 *^B^*	N (0.117)/M	N (0.116)/W	W (0.129)/M	W (0.124)/W
*A. butzleri* UPa 2015/1 *^B^*	W (0.129)/M	W (0.132)/W	N (0.118)/M	W (0.122)/W
*A. butzleri* UPa 2015/5 *^A^*	W (0.152)/M	W (0.202)/W	N (0.118)/M	W (0.121)/W
*A. butzleri* UPa 2015/6 *^B^*	W (0.125)/N	W (0.131)/N	N (0.117)/W	W (0.125)/N
*A. butzleri* UPa 2015/7 *^B^*	M (0.241)/M	W (0.170)/W	W (0.120)/M	W (0.126)/W
*A. butzleri* UPa 2015/9 *^B^*	W (0.127)/W	W (0.127)/W	N (0.120)/W	W (0.129)/W
*A. butzleri* UPa 2015/10 *^B^*	W (0.141)/W	W (0.140)/W	W (0.137)/M	W (0.138)/W
*A. butzleri* UPa 2015/11 *^B^*	W (0.134)/N	W (0.137)/N	W (0.176)/M	W (0.216)/W
*A. butzleri* UPa 2015/12 *^B^*	W (0.176)/N	W (0.135)/N	N (0.117)/N	W (0.122)/N
*A. butzleri* UPa 2015/13 *^B^*	N (0.120)/N	W (0.138)/N	W (0.139)/M	W (0.146)/N
*A. butzleri* UPa 2015/14 *^B^*	W (0.130)/W	W (0.136)/M	W (0.124)/M	W (0.136)/N
*A. butzleri* UPa 2015/15 *^B^*	W (0.177)/N	W (0.213)/N	W (0.142)/M	W (0.127)/N
*A. butzleri* UPa 2015/16 *^B^*	W (0.198)/M	M (0.375)/W	W (0.147)/M	W (0.217)/M
*A. butzleri* UPa 2015/18 *^B^*	W (0.131)/M	W (0.196)/W	N (0.120)/N	W (0.127)/W
*A. butzleri* UPa 2015/19 *^B^*	W (0.133)/N	W (0.134)/N	W (0.132)/M	W (0.149)/N
*A. butzleri* UPa 2015/20 *^C^*	W (0.139)/N	W (0.139)/N	W (0.128)/M	W (0.143)/N
*A. butzleri* UPa 2015/25 *^B^*	M (0.301)/W	W (0.160)/N	W (0.151)/M	W (0.146)/W
*A. butzleri* UPa KK *^B^*	N (0.109)/M	N (0.117)/W	N (0.119)/M	W (0.121)/M
*A. butzleri* UPa 24A *^B^*	W (0.138)/M	M (0.418)/M	W (0.169)/M	M (0.246)/M
*A. butzleri* UPa 30B *^B^*	W (0.131)/W	N (0.118)/N	W (0.137)/M	N (0.118)/W
*A. butzleri* UPa 39-3 *^C^*	W (0.143)/N	W (0.134)/M	M (0.242)/N	W (0.164)/N
*A. butzleri* UPa 49B *^B^*	W (0.130)/W	W (0.128)/N	W (0.124)/M	W (0.122)/W
*A. butzleri* UPa 65A *^B^*	W (0.130)/W	W (0.128)/W	W (0.128)/M	W (0.173)/M
*A. butzleri* UPa 132A *^B^*	W (0.127)/W	W (0.151)/W	N (0.113)/M	W (0.125)/M
*A. butzleri* UPa 138A *^B^*	W (0.141)/M	M (0.265)/W	W (0.122)/M	W (0.153)/W
*A. butzleri* UPa 141B *^B^*	W (0.123)/W	W (0.137)/W	N (0.116)/M	W (0.134)/M

*^A^* strain isolated from water; *^B^* strain isolated from food, *^C^* clinical isolate; UPa—strains isolated at University of Pardubice. N—non-adherent, W—weakly adherent, M—moderately adherent, S—strongly adherent. Value in parentheses represents the actual measured absorbance value.

**Table 2:** Biofilm formation of *A. cryaerophilus* strains (subgroups 1A and 1B) on plastic/glass material at 30 °C.

Strains	Exposure for 24 h		Exposure for 72 h	
	Aerobic	Microaerophilic	Aerobic	Microaerophilic
*A. cryaerophilus* 1B UPa 2012/1 *^A^*	W (0.130)/M	W (0.121)/W	W (0.126)/M	W (0.129)/W
*A. cryaerophilus* 1B UPa 2013/1 *^B^*	N (0.119)/N	W (0.127)/W	W (0.150)/M	W (0.143)/M
*A. cryaerophilus* 1A UPa 2013/12 *^A^*	N (0.119)/N	W (0.129)/N	W (0.146)/W	W (0.154)/W
*A. cryaerophilus* 1B UPa 2013/13 *^A^*	W (0.128)/M	W (0.165)/M	W (0.124)/W	W (0.134)/W
*A. cryaerophilus* 1B UPa 2013/14 *^A^*	W (0.136)/N	W (0.144)/W	W (0.133)/N	W (0.136)/N
*A. cryaerophilus* 1B UPa 2013/16 *^A^*	W (0.121)/M	W (0.123)/W	W (0.124)/W	W (0.129)/M
*A. cryaerophilus* 1B UPa 2013/17 *^B^*	N (0.118)/M	N (0.117)/W	W (0.127)/W	W (0.129)/M
*A. cryaerophilus* 1B UPa 2013/28 *^B^*	N (0.117)/M	W (0.122)/W	W (0.133)/M	W (0.137)/W
*A. cryaerophilus* 1B UPa 2013/35 *^A^*	W (0.130)/M	W (0.195)/M	N (0.118)/M	N (0.118)/W
*A. cryaerophilus* 1B UPa 2014/58 *^B^*	W (0.129)/W	W (0.122)/W	W (0.142)/M	W (0.145)/W
*A. cryaerophilus* 1B UPa 2014/58A *^B^*	W (0.128)/W	W (0.137)/W	W (0.155)/M	M (0.242)/M
*A. cryaerophilus* 1B UPa 2014/58D *^B^*	W (0.144)/N	W (0.137)/N	W (0.151)/M	W (0.177)/W
*A. cryaerophilus* 1B UPa 2014/59 *^B^*	W (0.127)/N	W (0.132)/M	W (0.147)/W	W (0.135)/N
*A. cryaerophilus* 1B UPa 130 *^B^*	W (0.125)/W	W (0.131)/N	W (0.121)/S	N (0.116)/M

*^A^* strain isolated from water; *^B^* strain isolated from food; UPa—strains isolated at University of Pardubice, N—non-adherent, W—weakly adherent, M—moderately adherent, S—strongly adherent. Value in parentheses represents the actual measured absorbance value.

**Table 3:** Biofilm formation of *Arcobacter*-reference strains on plastic/glass material at 30 °C.

Strains	Exposure for 24 h		Exposure for 72 h	
	Aerobic	Microaerophilic	Aerobic	Microaerophilic
*A. butzleri* CCUG 30484	W (0.152)/M	W (0.179)/W	W (0.178)/M	M (0.241)/W
*A. butzleri* LMG 10828	W (0.126)/M	W (0.130)/W	W (0.132)/M	W (0.178)/W
*A. cryaerophilus* 1B CCM 3933	N (0.118)/N	N (0.114)/M	N (0.120)/W	W (0.195)/N
*A. cryaerophilus* 1B CCM 7050	W (0.133)/M	W (0.138)/M	N (0.116)/W	W (0.130)/W
*A. defluvii* LMG 25694	W (0.228)/S	M (0.379)/S	M (0.414)/S	S (0.504)/S
*A. lanthieri* LMG 28517	W (0.125)/W	W (0.132)/N	W (0.123)/M	W (0.140)/N
*A. skirrovii* LMG 6621	W (0.126)/N	W (0.130)/N	W (0.136)/M	W (0.129)/M
*A. thereius* LMG 24488	W (0.124)/N	W (0.127)/N	N (0.120)/M	W (0.127)/W

CCUG—strains obtained from Culture Collection University Göteborg, LMG—strains obtained from the Belgian Co-ordinated Collections of Microorganisms, CCM—strains obtained from the Czech Collection of Microorganisms, N—non-adherent, W—weakly adherent, M—moderately adherent, S—strongly adherent. Value in parentheses represents the actual measured absorbance value.

**Table 4:** Biofilm formation of *Campylobacter* strains on plastic/glass material at 42 °C.

Strains	Exposure for 24 h		Exposure for 72 h	
	Aerobic	Microaerophilic	Aerobic	Microaerophilic
*Campylobacter jejuni* CCM 6214	W (0.121)/M	N (0.115)/M	N (0.093)/M	N (0.117)/M
*Campylobacter jejuni* NP 2896 *^C^*	W (0.126)/W	N (0.119)/W	N (0.092)/N	N (0.117)/W
*Campylobacter jejuni* NS 3668 *^C^*	W (0.125)/N	W (0.127)/N	N (0.114)/W	N (0.117)/W
*Campylobacter jejuni* NS 3800 *^C^*	W (0.121)/N	W (0.127)/W	N (0.115)/N	W (0.121)/N
*Campylobacter jejuni* NS 4088 *^C^*	W (0.124)/N	W (0.127)/N	N (0.119)/N	W (0.129)/N
*Campylobacter jejuni* NS 4091 *^C^*	W (0.124)/W	W (0.125)/N	N (0.113)/N	N (0.118)/N
*Campylobacter coli* NP 2395 *^C^*	W (0.123)/W	W (0.125)/N	N (0.118)/N	W (0.125)/N
*Campylobacter coli* NS 3803 *^C^*	W (0.121)/W	N (0.116)/N	N (0.094)/N	N (0.116)/N
*Campylobacter coli* NS 4062 *^C^*	W (0.121)/W	N (0.115)/N	N (0.094)/N	N (0.118)/W
*Campylobacter* sp. ONN 366 *^C^*	W (0.132)/W	W (0.130)/N	N (0.115)/N	N (0.118)/W

*^C^* clinical isolate; CCM—strains obtained from the Czech Collection of Microorganisms, NP—strains obtained from Pardubice Hospital, NS—strains obtained from Svitavy Hospital, ONN—strains obtained from Náchod Regional Hospital, N—non-adherent, W—weakly adherent, M—moderately adherent, S—strongly adherent. Value in parentheses represents the actual measured absorbance value.
